# A population-based study of synchronous distant metastases and prognosis in patients with PDAC at initial diagnosis

**DOI:** 10.3389/fonc.2023.1087700

**Published:** 2023-01-26

**Authors:** Leiming Zhang, Rong Jin, Xuanang Yang, Dongjian Ying

**Affiliations:** ^1^ Department of Minimally Invasive Surgery, The Affiliated Lihuili Hospital, Ningbo University, Ningbo, Zhejiang, China; ^2^ School of Medicine, Ningbo University, Ningbo, Zhejiang, China

**Keywords:** pancreatic cancer, distant metastasis, predictor, nomogram, prognosis factors

## Abstract

**Objective:**

Cancer of the pancreas is a life-threatening condition and has a high distant metastasis (DM) rate of over 50% at diagnosis. Therefore, this study aimed to determine whether patterns of distant metastases correlated with prognosis in pancreatic ductal adenocarcinoma (PDAC) with metastatic spread, and build a novel nomogram capable of predicting the 6, 12, 18-month survival rate with high accuracy.

**Methods:**

We analyzed data from the Surveillance, Epidemiology, and End Results (SEER) database for cases of PDAC with DM. Kaplan-Meier analysis, log-rank tests and Cox-regression proportional hazards model were used to assess the impact of site and number of DM on the cancer-specific survival (CSS) and over survival (OS). A total of 2709 patients with DM were randomly assigned to the training group and validation group in a 7:3 ratio. A nomogram was constructed by the dependent risk factors which were determined by multivariate Cox-regression analysis. An assessment of the discrimination and ability of the prediction model was made by measuring AUC, C-index, calibration curve and decision curve analysis (DCA). In addition, we collected 98 patients with distant metastases at the time of initial diagnosis from Ningbo University Affiliated LiHuili Hospital to verify the efficacy of the prediction model.

**Results:**

There was a highest incidence of liver metastases from pancreatic cancer (2387,74.36%), followed by lung (625,19.47%), bone (190,5.92%), and brain (8,0.25%). The prognosis of liver metastases differed from that of lung metastases, and the presence of multiple organ metastases was associated with poorer prognosis. According to univariate and multivariate Cox-regression analyses, seven factors (i.e., diagnosis age, tumor location, grade of tumor differentiation, T-stage, receipt of surgery, receipt of chemotherapy status, presence of multiple organ metastases) were included in our nomogram model. In internal and external validation, the ROC curves, C-index, calibration curves and DCA were calculated, which confirmed that this nomogram can precisely predict prognosis of PDAC with DM.

**Conclusion:**

Metastatic PDAC patients with liver metastases tended to have a worse prognosis than those with lung metastases. The number of DM had significant effect on the overall survival rate of metastatic PDAC. This study had a high prediction accuracy, which was helpful clinicians to analyze the prognosis of PDAC with DM and implement individualized diagnosis and treatment.

## Introduction

The mortality rate of all cancer has decreased significantly with progress in medicine and health care, save for one. As per the latest report of American Cancer Statistics in 2022 published by the ACS (American Cancer Society) (1), pancreatic cancer remains elusive to treatment and is emerging as the third leading cause in terms of mortality for men and women combined, and it will become the second leading cause of cancer death, according to some estimates (2, 3). Due to the rich supply of blood and lymph vessels in the pancreas, the cells of pancreatic cancer can easily invade large blood vessels and nerves surrounding the organ (4). This is why the vast majority of patients (over 50%) diagnosed with pancreatic cancer have distant metastasis even when the tumor is small (5, 6). Based on 2008-2014 reports, the stage distribution for selected cancers reported being confined to the metastasis (47%), followed by regional stage (29%), primary site (13%) and unstaged (11%) (1). And the percentage of five years survival rate is lowest in people with cancer in the metastasis (3%), followed by regional (33%), and localized (64%), based on patients selected from 2011 to 2017 (1). Metastatic pancreatic cancer has the worst prognoses and only limited progress has been made in revealing the relationship between pancreatic cancer metastasis and prognosis.

Based on previous studies, the liver is the organ most commonly affected by metastatic disease of pancreas, followed by the lungs, abdominal lymph nodes, peritoneum/omentum, bone and adrenal glands (7). In 90% of patients with pancreatic cancer, distant metastases are discovered at autopsy (8). Thus, it shouldn’t come as any surprise that nearly all patients with pancreatic cancer die from metastatic cancer (9). In our knowledge, however, there are few studies providing reliable insights into the relationship between metastatic pattern and pancreatic cancer prognosis, and neither a predictive model nor a prognostic model was derived for pancreatic cancer with DM. Pancreatic ductal adenocarcinoma (PDAC) is the most common pathological type of pancreatic tumor, therefore, evaluating the prognosis of PDAC with DM and constructing accurate models to predict risks are urgent and imperative.

The nomogram, a prediction model that can intuitively quantify the likelihood of specific events of interest, which transforms traditional statistical predictive models into visualized probability estimates tailored to each patient, is currently widely used in the prediction of clinical efficacy of various diseases (10–12). Our study goal is to investigate the effect of different site of the metastasis, number of sites and other relevant factors on patient prognosis, identifying a representative cohort from the Surveillance Epidemiology, and End Results (SEER) database. Finally, a nomogram was established to facilitate potential clinical applications for PDAC patients with DM.

## Materials and methods

### Data source

The data of metastatic PDAC patients currently studied were extracted from the SEER database between January 1, 2010, and December 31, 2015. SEER is supported by the Surveillance Research Program (SRP) in NCI’s Division of Cancer Control and Population Sciences (DCCPS), providing information on cancer statistics in an effort to reduce the cancer burden in the entire human population. Institutional Review Board approval was not required for any of the data because they were publicly available. An enormous, population-based cancer registry like SEER is able to extrapolate results to a broader population than single-center studies because more generalizable data of patients are available.

In addition, a total of 98 patients with initial diagnosis of metastatic PDAC were collected from Ningbo University Affiliated LiHuili Hospital, China, from October 1, 2017 to October 31, 2019.

### Inclusion and exclusion criteria

The following criteria were used to determine inclusion: (1) The primary cancer sites were classified according to the 3rd edition of the International Classification of Diseases in Oncology (i.e., ICD-O-3: C25.0-Head of pancreas, C25.1-Body of pancreas, C25.2-Tail of pancreas, C25.3-Pancreatic duct, C25.7-Other specified parts of pancreas, C25.8-Overlapping lesion of pancreas, C25.9-Pancreas). (2) Retrieval was restricted to patients with pathology of ICD-O-3 histology/behavior codes of 8140/3 (Adenocarcinoma), 8500/3 (Infiltrating duct carcinoma). (3) All the patients were diagnosed as stage IV by 7th edition of the American Joint Committee on Cancer (AJCC, 7th edition). These criteria were used for exclusion: (1) All individuals without microscopically confirmed pancreatic malignancy were excluded. (2) Those with a first malignancy other than pancreatic cancer were excluded. (3) Patients with unknown distant metastatic sites and whose tumor grade, race, or survival time were unavailable were also excluded. Furthermore, the dataset was omitted from patients with unknown surgery and adjuvant chemotherapy status. (4) Autopsies and death certificates were excluded from the study.

These criteria established our present study, 2709 patients were diagnosed as metastatic PDAC, including 2260 diagnosed with single organ DM. The 2260 patients were analyzed to determine if the site of DM was associated with the prognosis of patients, and all study subjects were randomly selected to participate in the training set (70%) and the validation set (30%) to explore the impact of the number of distant metastatic sites and other relevant factors on the survival time of patients with metastatic PDAC.

### Variables collected

The following parameters were collected after identifying cases for inclusion in this study: age at diagnosis (<50/50~64/>65), sex (Male/Female), ethnicity (White/Black/Other), primary site of the tumor (Pancreatic Head/Body-Tail/Other), tumor differentiation grade ((I-II/III-IV), pathological primary tumor T-Stage according to AJCC 7th ed (T1/T2/T3/T4), receipt of surgery (Yes/No), receipt of chemotherapy status (Yes/No or Unknown), the sites of distant metastases (Liver/Lung/Bone or Brain), number of distant metastatic sites (1/>1), survival status (Alive/Dead), cause of death (Pancreatic/Other), and survival time in month (i.e., a period of time from the diagnosis of a disease to death).

### Statistical methods

The cancer-specific survival (CSS) rate is calculated after excluding those who have died from causes other than cancer. PCSS (pancreas cancer-specific survival) is defined as death due to pancreatic cancer. In the study cohort of 2260 patients with one site of DM, the hazard ratio (HR) and associated 95% confidence intervals (CIs) for PCSS and OS were examined using Cox-proportional hazards regression model by univariable analysis. The comparison of OS and CSS among groups with different metastasis sites was conducted using Kaplan-Meier method with log-rank test. The 2709 PDAC patients were randomly divided into training and validation sets in a ratio of 7:3 by R software. All hypothesis tests were 2-sided with a significance level of 0.05.

In the entire study cohort, a multivariate analysis of the variables with *p <*0.05 in the univariate analysis was carried out (i.e., Cox-proportional hazards regression model). We also performed subgroup analysis and used forest plots to show HR values for DM patterns in specific subgroups. The comparison of OS and CSS between groups with or without multiple sites of DM was also conducted using Kaplan-Meier method with log-rank test. Besides, nomogram was created by running the R-package rms in R software by integrating variables with *p <*0.05 in multivariate analysis to form a personalized prediction model. The model was retested for internal validation using bootstrap, with 1000 bootstrap replicates, and the established nomogram was then calibrated using calibration curves and assessed by concordance indexes (C-index), area under receiver operating characteristic curves (AUC) and decision curve analysis (DCA). Observed results were compared to predicted probabilities using calibration curves. Generally, models with C-indices closer to 1 have improved predictive power. Similarly, nomograms with a calibration curve that is more closely aligned with the 45° diagonal have better predictive ability. Additionally, we applied 98 Chinese patients meeting the above criteria as external cohort to validate the effectiveness of this prediction model.

## Results

### Characteristics of the study population at baseline

Between 2010 and 2015, this study included 2709 patients with metastatic PDAC based on the criteria described above. Most patients (2260,83.43%) had single-organ DM and were listed separately as a study cohort. In addition, all patients were divided into a training set and a validation set, regardless of whether they had single-organ metastases or multi-organ metastases. As shown in **Table 1**, the demographic and clinicopathological characteristics of study patients are presented. Training and validation sets were mostly composed of older people >65 years of age (59.0% in the training set and 59.5% in the validation set), primary pancreatic tumors occur most often in the head of pancreatic tissue (49.5% in the training set and 44.9% in the validation set), followed by pancreatic body and tail (38.0% in the training set and 41.2% in the validation set). As for tumor grading, grading of tumor differentiation was most common in grades III-IV (55.5% in the training set and 54.9% in the validation set). As for the treatment strategies for PDAC with DM at initial diagnosis, 90 patients (3.32%) were treated with the aggressive surgical treatment alone, 160 (5.90%) with surgery combined with adjuvant chemotherapy, 1460 (53.89%) with adjuvant chemotherapy alone. Approximately, 10.40% of patients with single metastatic site were treated surgically, significantly higher than those with multiple metastases (15,3.34%). The Chi-square test showed that the deviations were purely random. In addition, **Table 2** demonstrates the demographic and clinicopathological characteristics of 98 Chinese patients.

**Table 1:** Baseline clinical characteristics of metastatic PDAC patients.

Variable	Single site (N=2260)	Entire cohort (N=2709)		
		Training(N=1896)	Validation(N=813)	P
**Age(years)**				0.757
<50	115 (5.1%)	100 (5.3%)	37 (4.6%)	
50 ~ 64	818 (36.2%)	678 (35.8%)	292 (35.9%)	
≥65	1327 (58.7%)	1118 (59.0%)	484 (59.5%)	
**Sex**				0.13
Male	1232 (54.5%)	1049 (55.3%)	424 (52.2%)	
Female	1028 (45.5%)	847 (44.7%)	389 (47.8%)	
**Race**				0.355
White	1776 (78.6%)	1479 (78.0%)	652 (80.2%)	
Black	315 (13.9%)	272 (14.3%)	100 (12.3%)	
Other	169 (7.5%)	145 (7.6%)	61 (7.5%)	
**Tumor location**				0.088
Head	1127 (49.9%)	938 (49.5%)	365 (44.9%)	
Body/tail	847 (37.5%)	721 (38.0%)	335 (41.2%)	
Other	286 (12.7%)	237 (12.5%)	113 (13.9%)	
**Grade**				0.768
I-II	1025 (45.4%)	844 (44.5%)	367 (45.1%)	
III-IV	1052 (55.5%)	1052 (55.5%)	446 (54.9%)	
**Size of tumor (T stage)**				0.907
T1	69 (3.1%)	54 (2.8%)	25 (3.1%)	
T2	687 (30.4%)	570 (30.1%)	249 (30.6%)	
T3	934 (41.3%)	775 (40.9%)	321 (39.5%)	
T4	570 (25.2%)	497 (26.2%)	218 (26.8%)	
**Surgery**				0.112
No	2025 (89.6%)	1710 (90.2%)	749 (92.1%)	
Yes	235 (10.4%)	186 (9.8%)	64 (7.9%)	
**Chemotherapy**				0.732
No/Unknown	890 (39.4%)	758 (40.0%)	331 (40.7%)	
Yes	1370 (60.6%)	1138 (60.0%)	482 (59.3%)	
**Site of distant metastases**				–
Liver only	1957 (86.6%)	–	–	
Lung only	251 (11.1%)	–	–	
Bone/Brain only	52 (2.3%)	–	–	
**Number of sites of metastases**				0.866
1	–	1580 (83.3%)	680 (83.6%)	
>1	–	316 (16.7%)	133 (16.4%)	

For race, ‘other’ includes American Indian, AK Native, Asian, and Pacific Islander. For tumor location, ‘other’ includes overlapping lesion of pancreas, pancreatic duct, and other specified parts of pancreas.

**Table 2:** Baseline clinical characteristics of 98 Chinese patients.

Characteristic	N=98
Age	
<50	3 (3.1%)
50~64	45 (45.9%)
≥65	50 (51.0%)
Sex	
0	38 (38.8%)
1	60 (61.2%)
Grade	
I-II	35(35.7%)
III-IV	63 (64.3%)
Tumor Location	
Head	50 (51.0%)
Body/Tail	48(49.0%)
Tstage	
T1	0 (0%)
T2	27 (27.6%)
T3	37 (37.8%)
T4	34 (34.7%)
Surgery	
0	83 (84.7%)
1	15 (15.3%)
Chemotherapy	
0	27 (27.6%)
1	71 (72.4%)
Patterns of distant metastases	
Liver Only	42 (42.9%)
Peritoneum/Omentum Only	20 (20.4%)
Lung Only	7 (7.1%)
Bone Only	1 (1.0%)
Spleen Only	1 (1.0%)
Liver+Peritoneum/Omentum	13 (13.3%)
Liver+Lung	2 (2.0%)
Liver+Bone	2 (2.0%)
Liver+Adrenal	2 (2.0%)
Lung+Pelvis	1 (1.0%)
Peritoneum/Omentum+Adrenal	1 (1.0%)
Peritoneum/Omentum+Bone	1 (1.0%)
Liver+Adrenal+Spleen	1 (1.0%)
Liver+Peritoneum/Omentum+Spleen	1 (1.0%)
Liver+Lung+Bone+Adrenal	1 (1.0%)
Liver+Peritoneum/Omentum+Spleen+Colon	1 (1.0%)
Liver+Peritoneum/Omentum+Lung+Bone+Brain	1 (1.0%)

### Distribution of distant metastatic sites

The SEER database records 3210 distant metastasis sites in the 2709 patients. In consistent with previous reports, in most cases, PDAC metastasizes to the liver (2387,74.36%), followed by lung (625,19.47%), bone (190,5.92%), brain (8,0.25%). Most patients in this study were diagnosed with PDAC combined with single-organ metastases (2260,83.43%), followed by two sites (398,14.69%), and the proportion of three and four sites was 1.85% and 0.04%, respectively, as shown in **Table 3**. Liver metastases combined with lung metastases were the most common type of metastatic disease in patients with two distant sites (306,11.30%). **Table 3** shows the distribution of distant metastatic sites in greater detail.

**Table 3:** Patterns of distant metastases for the 2709 metastatic PDAC patients.

Sites of distant metastases	Overall (N=2709)
One site of distant metastasis	
Liver	1957 (72.2%)
Lung	251 (9.3%)
Bone	50 (1.8%)
Brain	2 (0.1%)
Two sites of distant metastasis	
Liver+lung	306 (11.3%)
Liver+bone	73 (2.7%)
Lung+bone	17 (0.6%)
Brain+bone	1 (0.0%)
Lung+brain	1 (0.0%)
Liver+brain	0 (0.0%)
Three sites of distant metastasis	
Liver+lung+bone	47 (1.7%)
Liver+lung+brain	2 (0.1%)
Liver+brain+bone	1 (0.0%)
Lung+brain+bone	0 (0.0%)
Four sites of distant metastasis	
Liver+lung+brain+bone	1 (0.0%)

### The impact of the metastatic site on CSS and OS

**Figure 1:**
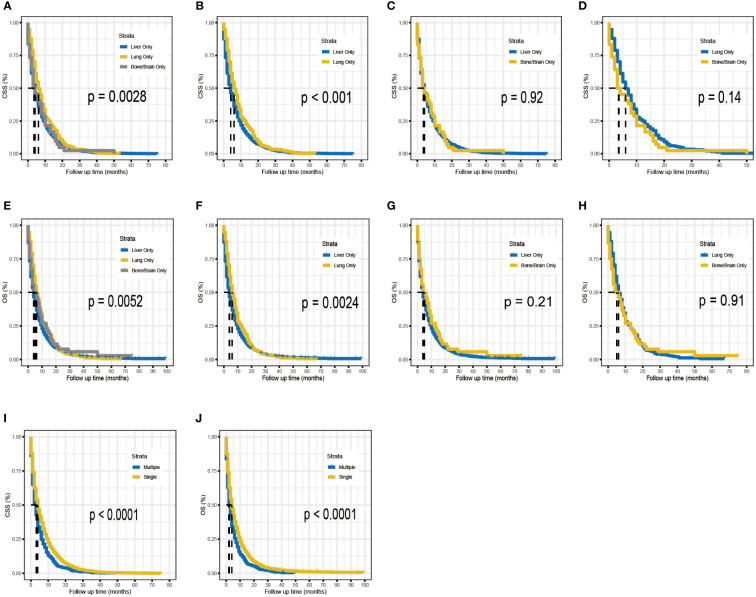
Kaplan–Meier analysis for CSS **(A–D)** and OS **(E–H)** of metastatic PDAC according to the metastatic site. Kaplan–Meier analysis for CSS **(I)** and OS **(J)** of metastatic PDAC according to the number of distant metastases.

**Figure 2:**
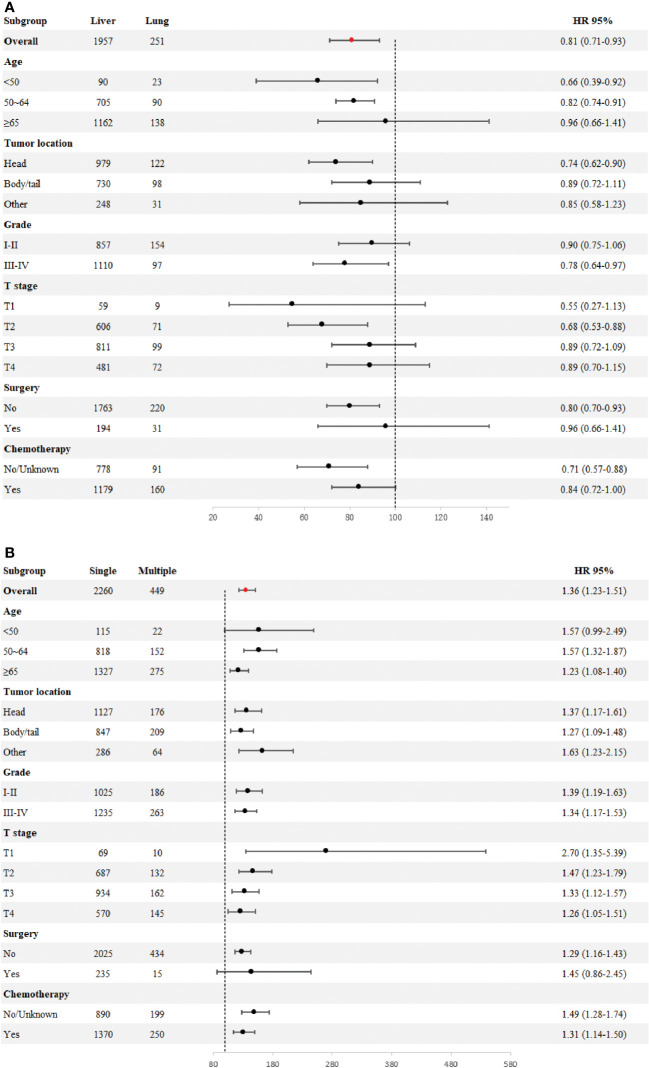
Forest plot for subgroup analysis of the sites of distant metastases **(A)**. Forest plot for subgroup analysis of the number of distant metastases **(B)**.

To determine if the metastatic site was a robust prognostic factor, we analyzed 2260 eligible patients with single-site DM. A total of 2040 patients died of PDAC, and the causes of death other than PDAC were competitive risk events. The univariate Cox regression analysis indicated that, using liver metastases as the reference, there was a longer survival rate among patients with lung metastases (for CSS: HR, 0.79; 95% CI, 0.69-0.90; *p* =0.0006)(for OS: HR, 0.81; 95% CI, 0.71-0.93; *p* =0.002). (**Table 4**). Meanwhile, Kaplan-Meier survival analyses were carried to compare CSS and OS among patients with different metastatic sites (**Figures 1A–H**). For CSS, the median survival time was 4, 6, and 3.5 months for patients with liver, lung, bone/brain metastases, respectively (liver vs lung metastases: *p <*0.001; liver vs bone/brain metastases: *p* =0.92; lung vs bone/brain metastases: *p* =0.14). For OS, the median survival time was 4, 6, and 5 months for patients with liver, lung, bone/brain metastases, respectively (liver vs lung metastases: *p* =0.002; liver vs bone/brain metastases: *p* =0.21; lung vs bone/brain metastases: *p* =0.91). The effect of the sites of DM on overall survival by subgroup is detailed in **Figure 2A**.

**Table 4:** Univariate Cox-regression analysis of the site of DM for cancer-specific survival and overall survival.

Variable	PCSS			OS		
	HR	95% CI	P	HR	95% CI	P
**Site of distant metastasis**						
Only liver	1			1		
Only lung	0.79	0.69-0.90	0.0006	0.81	0.71- 0.93	0.002
Only bone/brain	0.98	0.72-1.33	0.91	0.83	0.63-1.10	0.20

### Prognostic factors for metastatic PDAC

Across the entire cohort, single-site and multiple-site DM patients had a median survival time of 4 (95% CI, 4-4) and 3 (95% CI, 2-3) months, respectively (*p <*0.0001; **Figures 1I, J**). Besides, data regarding age at diagnosis, sex, ethnicity, primary site of tumor, tumor differentiation grade, size of tumor, receipt of surgery, receipt of chemotherapy, and number of sites of metastases were included in the multivariate Cox-regression analysis, which revealed that higher age (*p <*0.001), black race (*p* =0.049), tumors located in the body/tail (*p* =0.016), higher tumor grade and size (*p <*0.001), absence of surgery (*p <*0.001), absence of chemotherapy (*p <*0.001) and multiple sites of DM were significant prognostic factors for metastatic PDAC. Using one site of DM as the reference, there was a shorter overall survival rate among patients with multiple sites of DM (HR, 1.36; 95% CI, 1.23-1.51; *p <*0.001). Considering the limitations of univariate analysis, multivariable Cox-analyses were carried out to identify independent prognostic factors. Our findings showed longer survival benefit after aggressive surgical treatment and adjuvant chemotherapy. However, race did not appear to be an independent prognostic factor in multivariate Cox-regression, contrary to the result of univariate Cox-regression (**Table 5**). Subgroup comparisons for overall survival of age, tumor location, tumor grade, T-Stage, surgery and chemotherapy are shown in **Figure 2B**. Poorer prognosis was found in the multiple sites of DM group, in every subgroup analysis of overall survival, compared with the one site of DM group. Multiple metastases did not have a statistically significant prognosis for patients with PDAC in the <50 years age group (115 patients in the one site of DM group, median overall survival 8 months, 95% CI 7–10; 22 patients in the multiple sites of DM group, median overall survival 3.5 months, 95%CI 2–11; HR, 1.57; 95% CI, 0.99–2.49), Multiple metastases had a greater prognostic impact on PDAC patients in the 50~64 years age group (818 patients in the one site of DM group, median overall survival 5 months, 95% CI 4–6; 152 patients in the multiple sites of DM group, median overall survival 3 months, 95%CI 2–4; HR,1.57; 95% CI, 1.32-1.87) than in the ≥ 65 years age group (1327 patients in the one site of DM group, median overall survival 3 months, 95% CI 3-4; 275 patients in the multiple sites of DM group, median overall survival 2 months, 95%CI 2–3; HR, 1.23; 95% CI, 1.08-1.40). The effect of the number of DM on overall survival by subgroup is detailed in **Figure 2B**.

**Table 5:** Univariate and multivariate Cox-analyses of prognostic factors for overall survival in metastatic PDAC patients.

Variable	Univariate analysis			Multivariate analysis		
	HR	95%	p-Value	HR	95%	p-Value
Age(years)						
<50	1			1		
50 ~ 64	1.25	1.04-1.49	0.02	1.14	0.95-1.36	0.17
≥65	1.58	1.32-1.88	<0.001	1.35	1.12-1.61	0.001
Sex						
Male	1					
Female	0.98	0.91-1.06	0.65			
Race						
White	1			1		
Black	1.15	1.03-1.28	0.02	1.11	0.99-1.24	0.07
Other	0.98	0.85-1.13	0.79	0.91	0.79-1.05	0.20
Tumor location						
Head	1			1		
Body/tail	1.11	1.02-1.20	0.02	1.10	1.01-1.20	0.02
Other	1.11	0.99-1.26	0.08	1.08	0.95-1.21	0.23
Grade						
I-II	1			1		
III-IV	1.31	1.21-1.41	<0.001	1.37	1.26- 1.48	<0.001
T stage						
T1	1			1		
T2	1.30	1.03-1.64	0.03	1.27	1.01-1.61	0.05
T3	0.95	0.76- 1.20	0.68	1.08	0.85-1.36	0.51
T4	1.14	0.90-1.44	0.29	1.18	0.93-1.49	0.17
Surgery						
No	1			1		
Yes	0.51	0.44-0.58	<0.001	0.52	0.46-0.60	<0.001
Chemotherapy						
No/Unknown	1			1		
Yes	0.35	0.32-0.38	<0.001	0.33	0.31-0.36	<0.001
Number of sites of metastases						
1	1			1		
>1	1.36	1.23- 1.51	<0.001	1.31	1.18-1.45	<0.001

For race, ‘other’ includes American Indian, AK Native, Asian, and Pacific Islander. For tumor location, ‘other’ includes overlapping lesion of pancreas, pancreatic duct, and other specified parts of pancreas.

### Development and internal validation of prognostic nomogram

**Figure 3:**
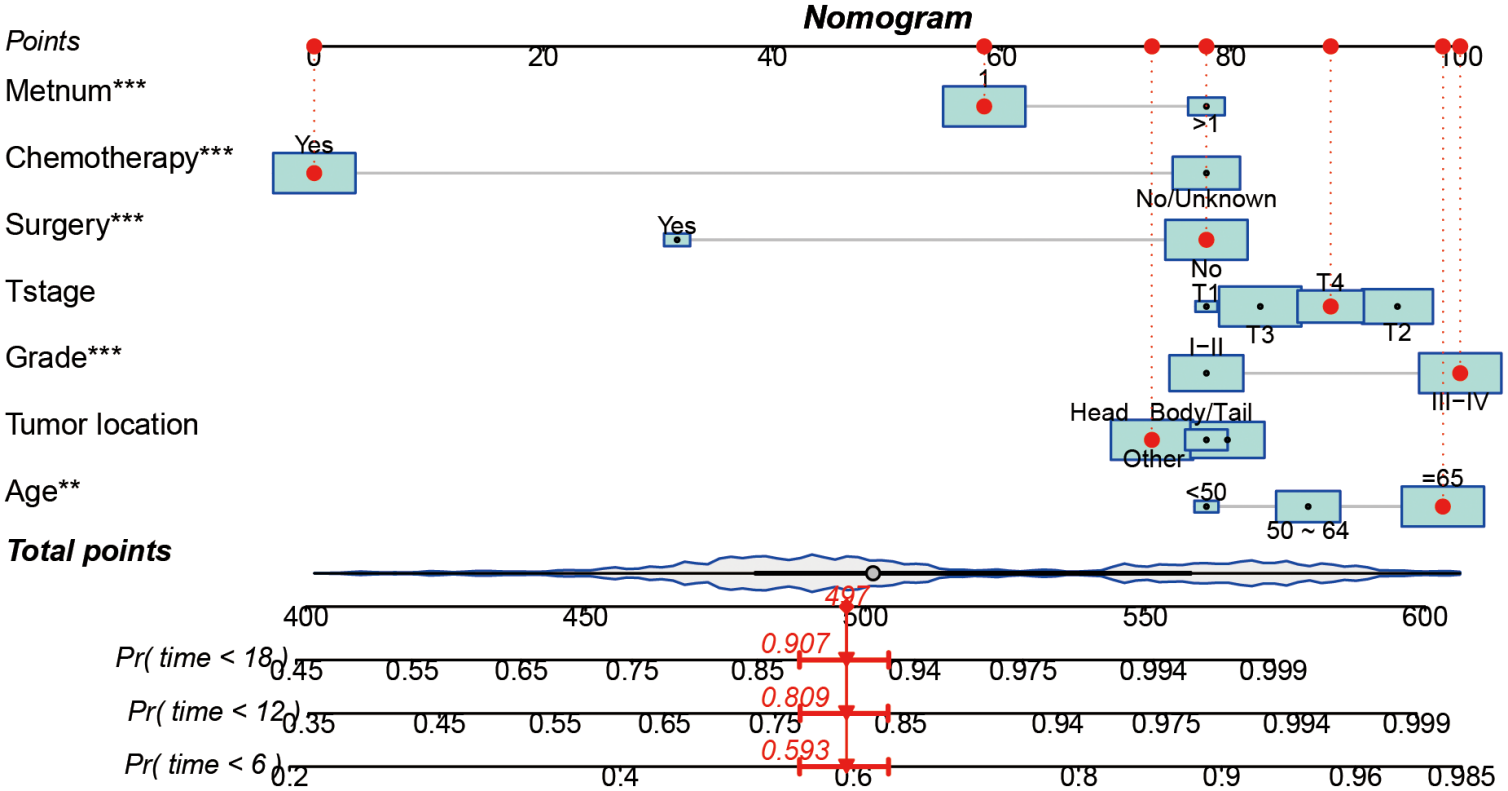
A prognostic nomogram for predicting the OS of metastatic PDAC patients for the 6, 12, and 18 months.

**Figure 4:**
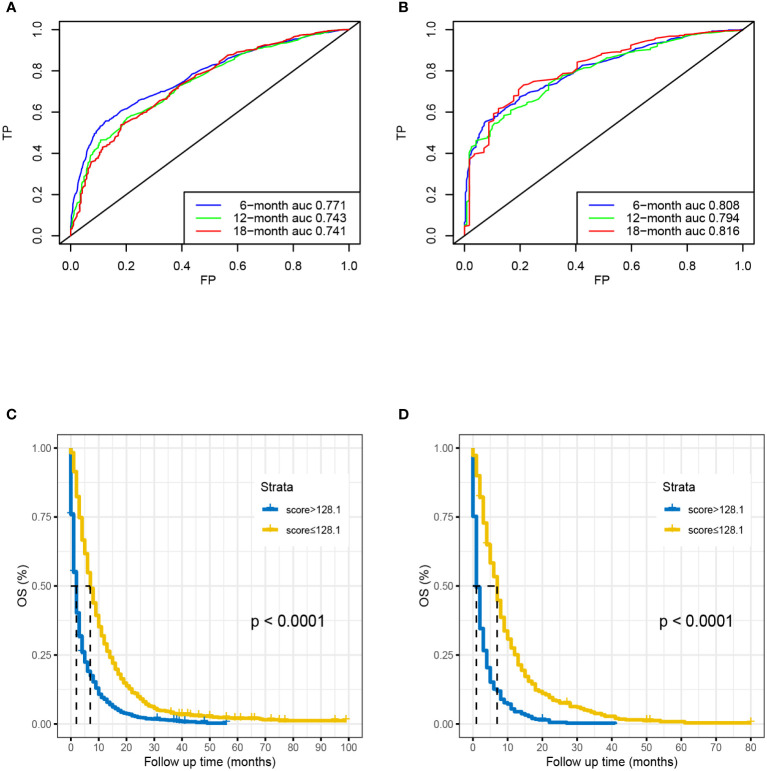
Time-dependent ROC curve analysis of the nomogram for the 6, 12, and 18 months in the training set **(A)** and the validation set **(B)**. The Kaplan–Meier survival curves of high-risk group and low-risk group in the training set **(C)** and in the validation set **(D)**.

**Figure 5:**
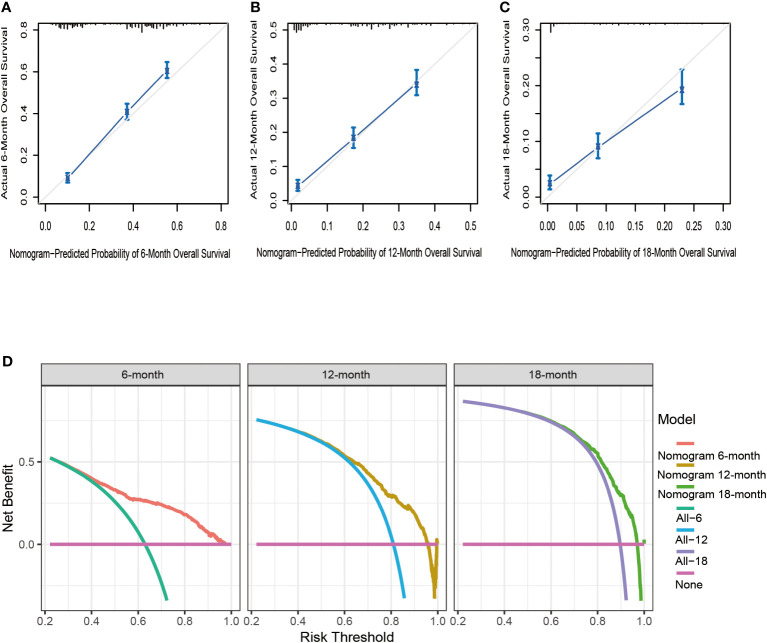
The calibration curves of the nomogram for the 6 **(A)**, 12 **(B)**, and 18 months **(C)** in the training set. The decision curve analysis of the nomogram for the 6, 12, and 18 months in the training set **(D)**.

**Figure 6:**
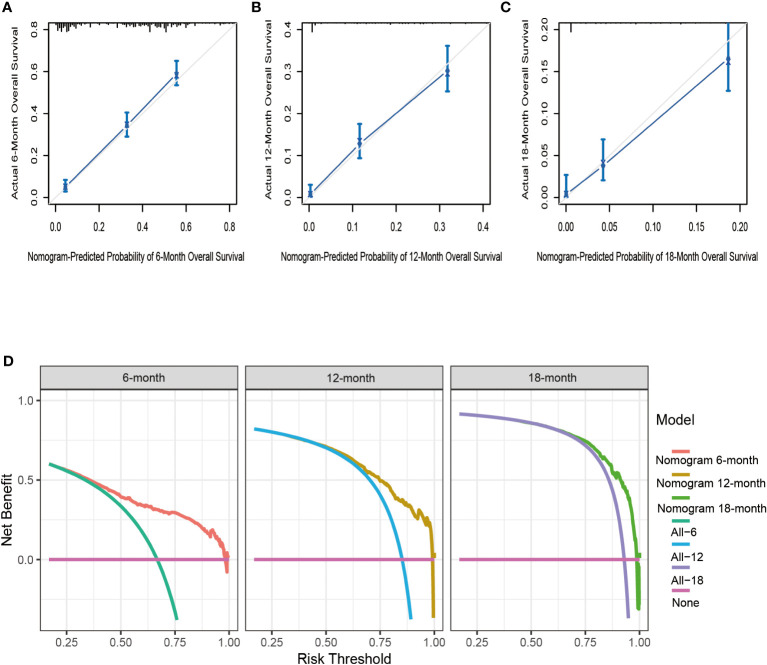
The calibration curves of the nomogram for the 6 **(A)**, 12 **(B)**, and 18 months **(C)** in the validation set. The decision curve analysis of the nomogram for the 6, 12, and 18 months in the validation set **(D)**.

Based on seven independent predictors, which are statistically significant in the multivariate Cox-regression analysis, a novel nomogram was developed for predicting OS in metastatic PDAC (**Figure 3**). In the next step, we established ROC curves for both training set and validation set, and the AUCs of the nomogram in the training set for the 6, 12 and 18 months reached 0.771, 0.743 and 0.741 (**Figure 4A**), and 0.808, 0.794 and 0.816, respectively, in the validation set (**Figure 4B**), indicating a high degree of prediction accuracy. Meanwhile, we calculated the total score of each patient according to nomogram and got the median number of 128.1 in the training set. Then we divided them into high-risk (total score > 128.1) and low-risk (total score ≤ 128.1) groups, and explored the survival difference between them by Kaplan Meier survival analyses (**Figures 4C, D**). More importantly, the C-indices for both training and validation sets were 0.713 and 0.735 respectively and survival predictions and actual observations were well matched by the calibration curves of this predictive model (**Figures 5A–C**, **6A–C**). Furthermore, DCA curves confirmed the clinical value of nomograms (**Figures 5D**, **6D**).

### External validation in 98 Chinese patients

**Figure 7:**
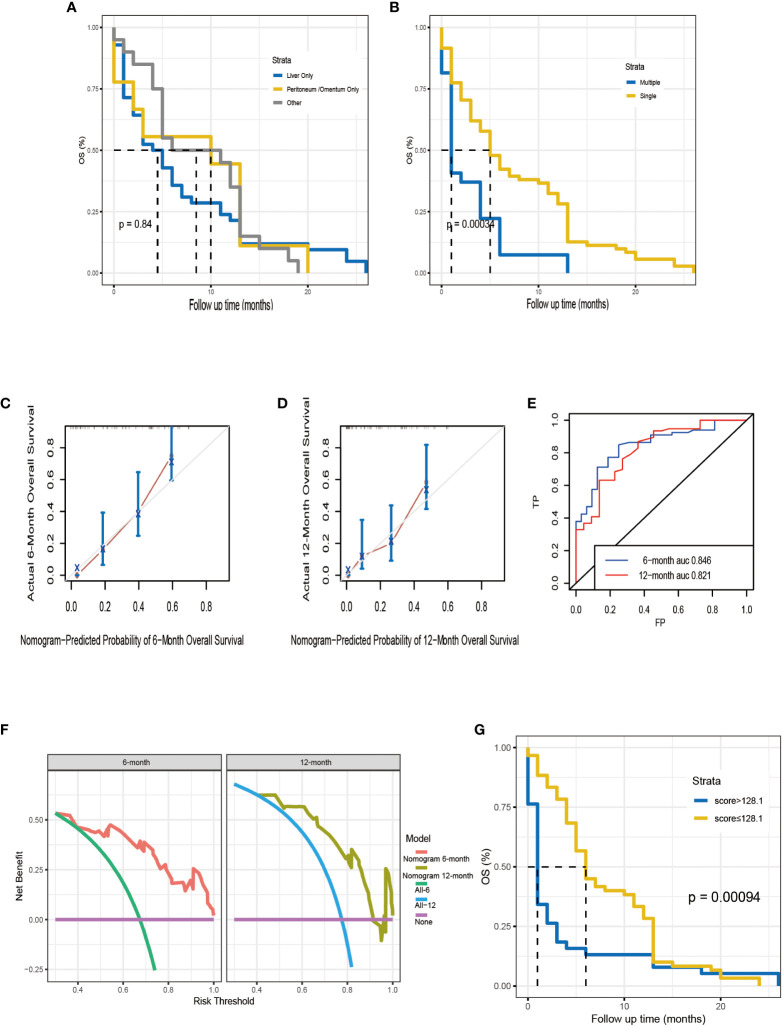
Kaplan–Meier analysis for OS of Chinese patients according to the metastatic site **(A)**. Kaplan–Meier analysis for OS of Chinese patients according to the number of distant metastases **(B)**. The calibration curves of the nomogram for the 6, 12 months in the external validation set **(C, D)**. Time-dependent ROC curve analysis of the nomogram for the 6, 12 months in the external validation set **(E)**. The DCA of the nomogram for the 6, 12 months in the external validation set **(F)**. The Kaplan–Meier survival curves of high-risk group and low-risk group in the Chinese cohort **(G)**.

Kaplan-Meier survival analyses were carried to compare OS among 98 Chinese patients with different metastatic sites. The median survival time was 4.5, 8.5, and 10 months for patients with liver, peritoneum/omentum, other (i.e., lung, bone, spleen) metastases, respectively (*p* =0.80; **Figure 7A**). Single-site and multiple-site DM patients had a median survival time of 5 (95% CI, 4-10) and 1 (95% CI, 1-4) months, respectively (*p <*0.001; **Figure 7B**). Due to the limitation of sample size, we only plotted the ROC curve, calibration curve and DCA for 6 months and 12 months (**Figures 7C–F**), and the C-index of external cohort was 0.752, indicating its high clinical predictive accuracy and clinical applicability. To further determine whether the nomogram is generalizable and reliable, we also divided this cohort into high-risk (total score > 128.1) and low-risk (total score ≤ 128.1) groups and explored the survival difference between them by Kaplan Meier survival analyses (**Figure 7G**). The results showed that high-risk (n = 38) and low-risk (n = 60) patients had a median survival time of 1 (95% CI, 1-2) and 6 (95% CI, 5-11) months, respectively (*p <*0.05). It follows that the established nomogram can be widely suggested.

## Discussion

Invasion and growth of tumors are key characteristics of pancreatic cancer, and more than 80% of patients with pancreatic cancer have already missed the chance to undergo surgical resection at the time of diagnosis (13, 14). The survival rate of patients sharply declines once the disease has metastasized (1). Research has increasingly focused on prognostic factors to predict pancreatic cancer survival and prognosis in recent years. However, their cohort studies were limited to individuals with early-stage or resectable carcinoma (15–19). Their summarized findings indicate that clinicopathologic factors such as resection margins status, perineural and blood vessel invasion, positive lymph nodes and perioperative blood transfusions are statistically significant prognostic variables. Nevertheless, above variables are unlikely to be available in patients with advanced pancreatic cancer who have lost access to surgery. Moreover, numerous studies focused on the molecular level rather than clinicopathologic features. H Friess et al. determined that *Bax*, a promoter of apoptosis, prolonged survival times in patients with pancreatic cancer by involving in the regulation of apoptosis (20). S. Dhara et al. conclude that PDAC could be predicted by ATAC-array (Assay for Transposase-Accessible Chromatin) (21). Jun Yu et al. found that high expression of *miR-200c* and *E-cadherin* was associated with a better prognosis of patients with pancreatic cancer (22). Fangfang Jin et al. also found that *tRNA*-derived small *RNAs* (*tsRNAs*) may serve as valuable biomarkers for predicting survival time of patients after surgery (23). However, it should be noted that these studies had low statistical power due to the sample size and study designs were single-center that should be used for further verification. More importantly, prognostic indicators at the molecular level are less convenient and practical than the easily available clinicopathological indicators. Thus, our data highlights the inclusion of clinicopathological factors in the prognostic assessment and our established prognostic model has more advantages in applying to clinical practice.

A MPACT (Metastatic Pancreatic Adenocarcinoma Clinical Trial) study revealed that the presence or absence of liver metastases were independent predictors of survival (24), and Josep Tabernero et al. reached the same conclusion. Subsequently, Renata D’Alpino Peixoto et al. found that in comparison to only local progression, development of peritoneal or distant metastases was associated with shorter OS (25). However, few studies have analyzed the connection between the patterns of DM and prognosis of metastatic PDAC. 2260 patients diagnosed with single DM were finally included for analysis the effect of different metastatic sites on the prognosis of metastatic PDAC in the present study. It has been demonstrated that the site of metastasis is a strong factor in the prognosis of certain metastatic diseases, such as ovarian cancer, prostate cancer, lung cancer, and testicular cancer (26–30). Our study suggested that metastatic PDAC with lung metastases had better survival outcomes (both CSS and OS) compared to metastatic PDAC with liver metastases. In addition, the number of DM sites also affected the survival outcome (both CSS and OS) of metastatic PDAC. The lung, as an organ with a rich blood supply, may be able to prolong the survival time of metastatic PDAC patients by buffering the invasion of metastatic cancer, even though patients with significant metastatic disease have a higher mortality rate due to organ failure or cachexia (31). It has been shown that metastatic PDAC patients with liver metastases have high levels of expression of epidermal growth factor receptor, E-cadherin and laminin, resulting in a tendency for multiple metastases and a poorer prognosis, which may be one of the reasons for the poorer prognosis of liver metastases compared with lung metastases (32).

Previous studies have shown that prognosis of pancreatic cancer may vary depending on several factors, including age, occupation, history of disease, location of the tumor, surgery method, perioperative complications, and TNM stage (33). In present study, we found that patients with the most advanced disease characteristic generally had the poorer prognosis (i.e., those diagnosed at a higher age, tumors located in the body/tail, higher tumor differentiation grade, larger tumor volume, presence of multiple metastatic sites), which is consistent with previous researches (34, 35). In the 2019 WHO Classification of Tumors of the Digestive System, the incidence of PDAC is about 85%, pancreatic neuroendocrine carcinoma (pNEC) account for about 5%, and other types of tumors account for the remaining 10% (36). The 5-year survival rate for PDAC is less favorable, at only 8% (36). Although the biological behavior and treatment of pNEC are very different from PDAC and often associated with a better prognosis, it has a significant risk of distant metastasis, even higher than PDAC. It has been mentioned in the literature that pNEC is often accompanied by metastasis, presenting with synchronous metastases in up to 65% of patients (37), aggressive treatment (surgery, radio-frequency ablation, etc.) will prolong the survival of patients with pNEC even in the presence of liver metastases (38, 39). Regarding the impact of PDAC location on patient prognosis, a previous study has found that cancers of the body and tail of the pancreas have a poorer prognosis. Previous studies evaluating a large cohort showed that less than 10% of patients had successful removal of pancreatic cancer from the body or tai (40, 41). Takeo Fujita et al. pointed out that these tumors in the body or tail become symptomatic much later than those in other areas such as the head of the pancreas, so that the tumors often cannot be removed due to extra-pancreatic involvement or distant metastases (42). In contrast, tumors located in the head of the pancreas induce obstructive jaundice at an early stage, which usually leads to seeking medical attention earlier, making them more curable and thus leading to a more favorable prognosis (43). As with previous findings, our research showed that tumors located in the head of the pancreas tended to have a better prognosis than the body of the pancreas, even when patients with PDAC had DM at the time of initial diagnosis.

Surgical resection of metastatic liver cancer has proven to be a treatment method that is effective for colorectal cancer (44) and gastric cancer (45), but no clear consensus has been reached on the treatment strategy of pancreatic cancer with simultaneous liver metastasis (46–48). However, surgeons continue to strive for longer survival time for patients with DM through aggressive surgical resection. A study of 69 participants with concurrent liver metastases showed that participants who underwent simultaneous resection had a longer survival time significantly compared to those who did not undergo surgical resection (14 months vs 8 months, *p* < 0.01*)* (49). An analysis of the SEER database suggests that CDC (cancer–directed surgery) is capable of prolonging survival of oligometastatic PDAC patients (50). In addition, locally-ablative treatments like microwave ablation (MWA) and radiofrequency ablation (RFA) are considered complementary to radical surgery (51). As for pancreatic cancer with lung metastases, a retrospective analysis of patients with pulmonary metachronous metastasis after PDAC collected from a database of two high-volume pancreatic cancer centers demonstrates that patients might benefit from surgical approaches of metachronous pulmonary metastasis (52). On the basis of the present data, it is also warranted that aggressive surgical treatment is associated with a better prognosis, but predictions of surgical outcomes for advanced pancreatic cancer with DM remain a challenge and many patients do not benefit from surgery.

In this study, chemotherapy remained one of the primary treatments for most patients with advanced pancreatic cancer and resulted in longer overall survival significantly. A MPACT, reported by von Hoff et al. (24) in 2013, is a phase III clinical trial of combined gemcitabine plus nab-paclitaxel therapy versus gemcitabine alone for first-line treatment in pancreatic cancer patients with DM. The findings indicated that the overall survival of patients receiving the combined regimen was significantly prolonged. In recent years, FOLFIRINOX regimen has also been recommended for the treatment of advanced pancreatic cancer with metastases (53).

Patients and their families rely on clinicians to predict the clinical course of their illness, and accurate predictions can prevent barriers to patient-doctor communication. To the present author’s knowledge, few studies have selected metastatic PDAC patients who lack effective treatment as study subjects, and there is still no prognostic prediction model developed for metastatic PDAC. Our study successfully developed a novel prognostic nomogram to predict the prognosis of metastatic PDAC, and this model was internally and externally validated to have an extremely high AUC, which demonstrated that this predictive model may provide a new source of clinical decision-making and personalized assessment. It is worth noting that there appears to be no logical order to T stage. It is easy to explain this: T stage is not a continuous variable and the recorded T stage was inaccurate because it is difficult to determine size measurements, and the distinction between T3 and T4 is now (AJCC, 7th edition) resectability, not size or invasion per se. Furthermore, note that we have not considered N stage for inclusion in our analysis. Because there are many factors affecting N stage, and the number of lymph nodes dissected is one of the main factors. The result of Valsangkar et al. (54) showed that predicting prognosis with N stage was ineffective when the number of lymph nodes dissected was less than 5 or 9. Strobel et al. (55) also found that the detection of more lymph nodes was more helpful to determine the N stage and predict the prognosis more accurately. Obviously, N stage was not applicable to the majority of patients, who missed the opportunity for radical surgical resection at the time of diagnosis.

We acknowledge that limitations exist with this study. First of all, the retrospective design of this study led many confounding variables and may render bias inevitable. Second, the limited number of pancreatic cancer patients with single brain/bone metastasis (N=52) may contributed to possible errors. Third, data from the SEER database do not provide information on disease progression because the information collected was about the disease at the time of initial diagnosis. Fourth, due to the limitations of the SEER database, only four site-specific distant metastases were included in our study. However, in addition to these sites, distant lymph nodes and peritoneum/omentum can also be metastasized in patients with metastatic pancreatic cancer (7), but the database we searched did not contain any detailed information about these distant metastatic sites. Although we further analyzed the prognostic differences between metastatic PDAC with hepatic and abdominal metastases, this study is a single-center retrospective study with its own limitations, and the relevant results still need to be further confirmed in a multicenter, large-sample randomized controlled trial. Additionally, detailed systemic treatment information, such as surgical modalities, palliative care, treatment regimens for adjuvant chemotherapy and their types, is not available.

## Conclusion

In summary, our study demonstrated that metastatic PDAC with lung metastases had better survival outcomes (both CSS and OS) compared to metastatic PDAC with liver metastases, and muti-organ metastases significantly shortened patient survival time. Notably, based on multivariate Cox-regression analysis, we developed a new nomogram, which can be used as a visual graphical tool for metastatic PDAC to quantitatively assess the risk and prognosis of metastatic PDAC and to guide clinical decision-making.

## Data availability statement

This study analyzed publicly available datasets. This data can be found here: https://seer.cancer.gov/data/.

## Ethics statement

The studies involving human participants were reviewed and approved by The Affiliated Lihuili Hospital, Ningbo University, Ningbo, Zhejiang, China. The ethics committee waived the requirement of written informed consent for participation.

## Author contributions

Study conception: LZ and DY, Data collection: LZ and RJ, Statistical analysis: LZ and XY, Article writing and revision: LZ and DY. All authors contributed to the article and approved the submitted version.
